# Age Trajectories of Perceptual Speed and Loneliness: Separating Between-Person and Within-Person Associations

**DOI:** 10.1093/geronb/gbab180

**Published:** 2021-11-09

**Authors:** Johanna Drewelies, Tim D Windsor, Sandra Duezel, Ilja Demuth, Gert G Wagner, Ulman Lindenberger, Denis Gerstorf, Paolo Ghisletta

**Affiliations:** 1 Department of Psychology, Humboldt University of Berlin, Berlin, Germany; 2 College of Education, Psychology and Social Work Flinders University, Adelaide, South Australia, Australia; 3 Max Planck Institute for Human Development, Berlin, Germany; 4 Charité–Universitätsmedizin Berlin, Berlin, Germany; 5 German Socio-Economic Panel Study (SOEP), Berlin, Germany; 6 Max Planck UCL Centre for Computational Psychiatry and Ageing Research, Berlin, Germany and London, UK; 7 University of Geneva, Geneva, Switzerland; 8 Swiss National Center of Competence in Research LIVES, University of Geneva, Geneva, Switzerland

**Keywords:** Age, Berlin Aging Study II, Cognition, Isolation, Old age

## Abstract

**Objectives:**

We aimed at examining between-person and within-person associations across age trajectories of perceptual speed and loneliness in old age.

**Method:**

We applied multilevel models to 4 waves of data collected over 6 years from 1,491 participants of the Berlin Aging Study II (60–88 years at baseline, 50% women) to disentangle between-person and within-person associations across age trajectories of perceptual speed and both emotional and social loneliness. Sex and education were considered as relevant individual characteristics and included as covariates in the model.

**Results:**

Analyses revealed that on average perceptual speed exhibited moderate within-person age-related declines, whereas facets of loneliness were rather stable. Perceptual speed did not predict age trajectories of emotional or social loneliness, at either the between- or within-person level. In contrast, loneliness discriminated individuals at the between-person level, such that those feeling emotionally or socially more lonely showed lower cognitive performance than those feeling emotionally or socially less lonely. Predictive effects of social loneliness were stronger for relatively young people (i.e., in their mid to late 60s) than for relatively older participants (i.e., in their 80s). In addition, predictive effects of social loneliness for perceptual speed at the within-person level were modest and deviated in direction and size from between-person social loneliness effects among those in their mid- to late 60s, whereas they did not among those in their 80s.

**Discussion:**

We conclude that loneliness may serve as a precursor for basic cognitive functioning in old age and suggest routes for further inquiry.

Life-span theory has long emphasized that cognitive functioning and avoiding loneliness are key constituents of successful development and aging (Rowe & Kahn, 1997). There is mounting empirical evidence that these central domains of life are closely intertwined, with better performance on a number of cognitive abilities relating to lower levels of loneliness (Cacioppo & Hawkley, 2009; Evans et al., 2019). Less well understood is, however, whether loneliness precedes cognitive functioning, and change therein, or whether, vice versa, low cognitive functioning operates as a risk factor for loneliness. Importantly, an increasing body of literature has suggested that associations among variables observed when making use of between-person information might not be equivalent to those found within individuals (e.g., Brose et al., 2014; Hamaker et al., 2007). For example, people who are cognitively fitter than others may report lower levels of loneliness than others (between-person association), but a person’s experience of cognitive decrements may not necessarily correspond with a rise in loneliness (within-person association).

The major objective of the present study is to examine the dynamic and reciprocal interplay between age trajectories of perceptual speed, assessed with the Digit Symbol (DS) test, and loneliness, and to separate predictive effects at the between-person level from those at the within-person level. We apply a series of multilevel models (MLMs) to four-wave, 6-year longitudinal data obtained from 1,491 older adults in the Berlin Aging Study II (BASE-II) and test empirically competing unidirectional accounts of such across-domain associations.

## Age Trajectories of Cognitive Functioning and Social and Emotional Loneliness in Old Age

Cognitive functioning can be conceived as a general-purpose mechanism for adaptation and a resource upon which people can draw when facing obstacles (Alwin et al., 2006). It is well known that age-normative cumulative decline in performance on tasks of broad fluid cognitive abilities such as perceptual speed commences in early adulthood (Schaie, 2005) and shows moderate-to-strong average declines in old age (Lindenberger & Ghisletta, 2009).

Loneliness can be defined as the subjective experience of being isolated (Peplau & Perlman, 1982). Conceptual notions have long distinguished between social and emotional facets of loneliness (Weiss, 1973). These are thought to arise from perceived discrepancies of one’s actual social network either with the desired quantity of one’s support network, such as friends, family, and neighbors (social loneliness [SL]), or with the desired quality of close emotional attachments and meaningful relationships (emotional loneliness [EL]). Both EL and SL have been shown to be crucial for one’s mental, physical, and cognitive health (Mund et al., 2020; Suanet & van Tilburg, 2019). Among other reasons, because prolonged loneliness might induce higher levels of stress, which, in the long run, may affect multiple aspects of health.

Empirical evidence on age-related change in EL and SL is equivocal. Some studies report age-related increases in SL (Suanet & van Tilburg, 2019), presumably because of reduced social contacts after retirement, widowhood, death of peers, and decreased physical functioning (von Soest et al., 2020). In contrast, other studies report relative stability, or only minor forms of increases in both SL and EL (Mund et al., 2020), presumably because older adults focus more on emotionally rewarding relationships, intensify and cherish such contacts, and thus remain emotionally close to others (Carstensen et al., 1999).

## The Interplay Between Age Trajectories of Cognitive Functioning and SL and EL

Several different conceptual accounts explain how cognitive functioning and loneliness may be intertwined. One position argues that cognitive functioning may serve as a general resource that individuals employ to master developmental challenges in ways that shape loneliness. To illustrate, impoverished cognitive functioning may act as a risk factor for increases in loneliness because it sets constraints on an individual’s capacity to engage in social activities, thereby contributing to social isolation (Düzel et al., 2019). Empirical findings support these notions, suggesting that levels of and changes in cognitive functioning are associated with subsequent loneliness in older adults. For instance, Okely and Deary (2018) analyzed longitudinal data from the Lothian Birth Cohort Study and concluded that individuals with lower cognitive abilities, such as perceptual speed, at age 73 years were at higher risk of becoming lonely over time than those with better abilities (see also Ellwardt et al., 2013).

Conversely, several conceptual positions argue that loneliness may serve as a risk factor for cognitive aging. One such position notes that people feeling lonely might have low exposure to sensory and cognitive stimuli, which may result in less enriched and stimulating environments (Wilson et al., 2007). Another position highlights the role of stress-regulatory processes, such that people who feel lonely experience more stress and heightened physiological stressor responses that increase the risk for cognitive decline and even dementia (Johansson et al., 2010). Again, much empirical evidence is in line with these notions (Luchetti et al., 2020; Shankar et al., 2017). For example, Zhong and colleagues (2017) examined 9-year longitudinal data from Chinese older adults and reported that more loneliness was associated with subsequent decrease in cognitive functioning, specifically Mini-Mental State Examination, and heightened risk for Alzheimer’s disease (see also Rafnsson et al., 2020).

## Separating Between-Person From Within-Person Associations

Differences between studies in design (e.g., duration), sampling strategy (e.g., old versus very old people), assessment approaches and measures (e.g., disease onset versus preclinical cognitive functioning), and cultural context (e.g., Western versus Eastern) may well have contributed to divergent findings. In the current study, we attempt to shed light onto the cognition–loneliness interplay by investigating another potential source of divergence. Specifically, the relevant empirical literature so far does not allow separating and disentangling associations that operate at the between-person level from those acting at the within-person level. Evidence from other fields indicates that findings obtained using between-person difference information do not necessarily generalize to the within-person level of analysis (Hoffman & Stawski, 2009). For example, if at a single point in time an individual reports not being lonely and having good cognitive functioning, and another person reports being both lonely and having poor cognitive functioning, this does not imply that either person will develop poorer cognitive functioning when experiencing being lonely (Curran & Bauer, 2011). Likewise, a between-person difference approach to longitudinal data allows us to demonstrate that, for example, people experiencing sharper cognitive decline than others are more likely to also feel more loneliness. Focusing on within-person associations, the task is to examine whether—within a given person—cognitive decrements over time are associated with increases in loneliness over time.

When analyzing only a single measurement occasion, it is not possible to disentangle those effects (Nesselroade, 1991). Data from longitudinal studies, on the other hand, contain information on both between-person and within-person variabilities. As a consequence, observable differences between people may either be due to static differences (between-person variability) or to time-related changes (within-person variability from one occasion to the next). By analyzing longitudinal data, we can disentangle between-person from within-person effects and thereby contribute to a more fine-grained examination of reciprocal associations between perceptual speed and loneliness among older adults (Thorvaldsson et al., 2012, 2020).

## The Present Study

The goal of the current study is to examine the interplay between age trajectories of cognitive functioning and loneliness, and separate between-person associations from within-person associations. To do so, we make use of MLMs applied to four-wave, 6-year longitudinal data obtained from 1,491 older adults in BASE-II. As a measure of cognitive functioning, we select perceptual speed, which is a descriptive term from the psychometric tradition (e.g., three-stratum theory as stated in Carroll, 1993) that accurately summarizes the variance captured by the DS test (Laux & Lane, 1985). This ability represents a powerful and highly sensitive proxy of cognitive decline in old age (Lindenberger & Baltes, 1997), loads highly on a factor of general intelligence (Tucker-Drob et al., 2014) and memory performance (e.g., MacDonald et al., 2008; Piccinin & Rabbitt, 1999; Salthouse, 1992), has excellent psychometric properties (Verhaeghen & Salthouse, 1997), and requires a sequence of perceptual, motor, and memory processes (Lindenberger et al., 1993).

As measures of loneliness, we consider both emotional and social facets. Empirically testing competing unidirectional accounts, we expect perceptual speed to be associated with age trajectories of loneliness and, vice versa, loneliness to be associated with age trajectories of perceptual speed, both at between-person and within-person levels. Conceptual and empirical work alike also suggests that multidomain associations might be moderated by third variables, such as sex and education. We thus control for sex and education, both of which have repeatedly been linked with cognitive functioning and loneliness (Gerstorf et al., 2006; von Soest et al., 2020). Specifically, women typically report being lonelier and tend to perform better on tests of processing speed compared to men (Borys & Perlman, 1985; Gerstorf et al., 2006). It thus appears conceivable that these two domains are more tightly connected in women relative to men. Relatedly, empirical work indicates that, in old age, fewer years of education are associated with poorer cognitive performance (Lövdén et al., 2020) and, in part, more loneliness (Hawkley et al., 2008), suggesting that across-domain associations may presumably be stronger at lower levels of education.

## Method

To examine our research questions, we used data from the BASE-II. Descriptions of participants, variables, and procedures are reported in previous publications (Bertram et al., 2014). Select details relevant for our report are provided below.

### Participants and Procedure

The BASE-II sample included residents of the greater metropolitan area of Berlin, Germany, recruited via a participant pool at the Max Planck Institute for Human Development Berlin (MPIB) and advertisements in local newspapers and the public transportation system. Because of our focus on old age, we analyzed only participants from the older subgroup aged 61–88 years (excluding the younger subgroup aged 20–35), 50% were female, and considered only those four timepoints with both cognitive and loneliness data (excluding the 2010, 2017, and 2019 timepoint that assessed only DS). Participants in our analysis sample were born between 1925 and 1953 and were initially interviewed and tested in 2012–2013. Ethics approval for BASE-II was granted by the ethics committees of the Charité–Universitätsmedizin Berlin and the MPIB.

The DS task was assessed by trained interviewers in groups with three to six participants in 2010 (T1; *n* = 1,246), 2012–2013 (T2; *n* = 1,430), 2012–2013 (T3; *n* = 1,462), 2016 (T4, with only a subset BASE-II sample measured on brain volume; *n* = 252), 2017 (T5; *n* = 82), 2018–2020 (T6; *n* = 860), and 2019 (T7; *n* = 901). Loneliness data were obtained either with take-home, paper-and-pencil questionnaires, or online in 2013 (T2; *n* = 1,492), 2014 (T3; *n* = 1,157), 2016 (T4; *n* = 255), and 2018–2020 (T6; *n* = 978). The large majority of participants contributed two or more data points on either time series (DS: 86%; loneliness: 88%, with 52.24% and 64.71% providing three or more measurement points on loneliness, respectively, DS), and thus lend themselves to the examination of within-person change. On average, individuals were observed across 3.67 years (*Median* = 4.85; *SD* = 2.32; range: 0–6.53 years) on the DS and 3.75 years (*Median* = 5.05; *SD* = 2.37; range: 0–6.26 years) on loneliness. Previous studies have identified associations between cognition and loneliness in older adults using sample sizes of approximately 500 participants (for review, see Mund et al., 2020). Using a sample size of over 1,000 in the current study will allow us to identify associations between the variables of interest. 

Sample selectivity analyses suggested that participants included in our analyses represent a positive selection of the larger population. For example, compared with BASE-II participants not included in our analyses (e.g., because of missing data on some of the relevant variables; *n* = 400), our participants were younger (*d* = 0.20) and more educated (*d* = 0.33). Likewise, compared with participants who completed only one assessment (*n* = 181), those who completed two or more assessments on loneliness (*n* = 1,350) were on average younger (*d* = 0.22), reported less EL (*d* = 0.23), and performed better on the DS test (*d* = 0.48). Our results may thus not generalize to more disadvantaged population segments.

### Measures

#### Digit Symbol

The Digit Symbol Substitution test (Wechsler, 1955) consists of a code box with nine digit–symbol pairs, and rows of double boxes with a digit in the top box and an empty lower box. Participants are asked to fill in as many corresponding symbols as possible in 90 s. We analyzed the number of correctly filled boxes, with penalty for wrong answers (score = correct – wrong).

#### Loneliness

We used seven items from the UCLA Loneliness Scale (Russell et al., 1984) to assess EL (e.g., “I lack companionship.”) and SL (e.g., “There are people I feel close to.”) with a 5-point Likert scale ranging from 1 = *does not apply to me at all* to 5 = *applies very well to me*. Items were coded such that higher scores mean higher loneliness. Internal consistencies (at T1, Cronbach’s α = .70 and α = .84, respectively) reflect the brief nature of the scales and the purposeful use of heterogeneous items across the construct space. We applied MLMs to obtain the within-person reliability estimates of EL and SL following the procedure suggested by Nezlek (2017). For SL, within-person reliability was .61 (higher occasion-level variance at .14 for less item-level variance at .36). For EL, it was lower, with .07 (little occasion-level variance at .02, compared to high item-level variance at .67; Nezlek, 2017). This was most probably a consequence of no variance in within-person age effects (cf. “Results” section).

#### Sociodemographic covariates

Our statistical models covaried for potentially relevant individual characteristics, including chronological age, sex (women = 1, men = 0), and education (number of years necessary to obtain the final school degree).

### Data Preparation and Data Analyses

Scores for the DS, EL, and SL were T-standardized using baseline data (*M* = 50; *SD* = 10). MLMs were estimated with SAS (Proc Mixed; Littell et al., 1996). Incomplete data were accommodated under usual missing at random assumptions (Little & Rubin, 1987), with included variables (age, sex, cognition) serving as attrition-informative variables that alleviate longitudinal selectivity for the outcome variables (Grimm et al., 2016). Descriptive statistics for the three outcomes by age are reported in Supplementary Table 1. The bulk of the data were obtained when our participants had been in their late 60s to early 80s.

In the MLM framework used, repeated assessments at Level 1 are nested within individuals at Level 2 and allow estimation of both between-person (Level 2) and within-person (Level 1) effects. We used the classical MLM centering strategy of covariates to orthogonalize between- and within-person effects (Raudenbush & Bryk, 2002). Our main covariates were age and either the cognitive marker (DS) or one of the loneliness variables (EL and SL). When studying cognitive performance, we carried out two models, each including age effects and either EL or SL effects. When studying EL or SL, we included age and the DS as main predictors. All models controlled for sex and education as potentially confounding covariates.

Disaggregating between-person and within-person effects within the MLM framework entails centering covariates that are repeatedly assessed in time because these carry information about both between-person differences (e.g., some participants are overall older than others) and within-person changes (e.g., participants get older across repeated assessments). For any outcome *Y*_*it*_, for individual *i* at age *t*, the full MLM is shown in Equation 1. First, for each individual *i*, we calculated their mean on the covariates to be centered across their repeated assessments. For instance, for every individual, we calculated the mean value of their repeated age values. This person-specific mean value is thus a between-person predictor, which we call *bpAge*_*i*_. Then, we subtracted this person-specific mean value from each person’s repeated assessments, obtaining a centered Level 1 predictor estimating within-person effects. For instance, for every individual, we subtracted *bpAge*_*i*_ from their repeated age values, and called the resulting differences *wpAge*_*it*_. Thus, we estimated between-person age effects via *bpAge*_*i*_, and within-person age effects via *wpAge*_*it*_. To keep the interpretation of the overall intercept, we centered the person-specific means *bpAge*_*i*_ on the overall sample mean. We applied the same procedure to the other covariate *X*_*it*_, whose between- and within-person effects we wanted to disaggregate, namely EL or SL when predicting DS, and DS when predicting EL or SL.

We also included sex (*W*_*i*_) and education (*E*_*i*_) and their interactions with both between- and within-person effects of *Age* and *X*. We centered *E*_*i*_ around the overall sample mean.


$$\begin{array}{l} \begin{array}{ll} & Y_{it}=\gamma_{00}+\gamma_{10}wpAge_{it}+\gamma_{01}bpAge_{i}+\gamma_{11}wpX_{it}+\\ & \gamma_{02}bpX_{i}+\gamma_{12}wpAge_{it}wpX_{it}+\gamma_{13}wpAge_{it}bpX_{i}\\ & +\gamma_{14}bpAge_{i}wpX_{it}+\gamma_{03}bpAge_{i}bpX_{i}+\gamma_{04}W_{i}+\gamma_{15}wpAge_{it}W_{i}\\ & +\gamma_{05}bpAge_{i}W_{i}+\gamma_{16}wpX_{it}W_{i}+\gamma_{06}bpX_{i}W_{i}+\\ & \gamma_{07}E_{i}+\gamma_{17}wpAge_{it}E_{i}+\gamma_{08}bpAge_{i}E_{i}+\gamma_{18}wpX_{it}E_{i}\\ & +\gamma_{09}bpX_{i}E_{i}+u_{0i}+u_{1i}wpAge_{it}+r_{it} \end{array} \end{array}$$ (1)

We allowed for individual-specific random effects around the intercept (*u*_0i_) and the slope of age (*u*_1i_) and included the time-specific residuals (*r*_*it*_). For similar applications, see Thorvaldsson and colleagues (2012, 2020).

## Results

We report descriptive statistics and intercorrelations among the measures of interest in Table 1. At baseline, the DS was not correlated with either of the two loneliness facets. In line with earlier reports, emotional and social facets of loneliness are interrelated (*r* = .54), yet capture different aspects of the larger concept space. Age, sex, and education are in multifaceted ways associated with the three measures of interest (thus are meaningfully included in our analyses).

**Table 1.** Descriptive Statistics at Baseline Assessment and Intercorrelations for Study Measures

			Intercorrelations					
	M	SD	1	2	3	4	5	6
(1) Digit Symbol (12–88)	49.71	10.51	1					
(2) Emotional loneliness (42–94)	50.03	10.04	–.04	1				
(3) Social loneliness (42–101)	50.01	10.12	–.05	**.54**	1			
(4) Age (61–88)	70.63	3.83	**–.08**	**.05**	**.06**	1		
(5) % Women	50.18		**.16**	.01	**–.09**	.01	1	
(4) Education (7–18)	14.55	3.03	**.13**	–.03	**–.07**	–.02	**–.18**	1

*Notes*: *M* = mean. *N* = 1,425. Scores for the Digit Symbol, emotional loneliness, and social loneliness were standardized to the T metric using the cross-sectional Berlin Aging Study II sample at T1 (*M* = 50, *SD* = 10). Intercorrelations in bold differ statistically significantly from zero at *p* < .05.

Table 2 reports parameter estimates and standard errors from the four models estimated. Beginning with the fixed effects, we see statistically significant decline on the DS at the within-person level (w-p age: –0.49/–0.48), but no significant effects of age at the between-person level (b-p age). This suggests that, after accounting for all other effects, older participants in our sample did not perform worse on the cognitive functioning test than younger adults, but participants deteriorate in cognitive performance by almost half a standard deviation across a 10-year epoch [–0.49/–0.48*10 = –4.9/–4.8]. In contrast, both loneliness facets are on average stable, with only modest evidence of within-person increases in EL (0.28, for a graphical representation, see Supplementary Figure 1). Moreover, for both loneliness aspects, random effects of within-person age were not significant, indicating no heterogeneity in within-person change in loneliness.

**Table 2.** Growth Models of Emotional Loneliness (EL), Social Loneliness (SL), and the Digit Symbol

	Emotional loneliness		Social loneliness		Digit Symbol		Digit Symbol	
	Est.	SE	Est.	SE	Est.	SE	Est.	SE
Fixed effects								
Intercept (γ_00_)	50.82**	0.41	51.03**	0.42	46.37**	0.47	46.51**	0.47
*wpAge* (γ _10_)	0.28*	0.12	0.02	0.14	–0.49**	0.12	–0.48**	0.12
*bpAge* (γ _01_)	–0.03	0.09	0.05	0.09	–0.13	0.09	–0.13	0.09
Digit Symbol								
*wpDS* (γ _11_)	–0.08	0.05	–0.12	0.06	—	—	—	—
*bpDS* (γ _02_)	–0.02	0.04	–0.06	0.04	—	—	—	—
*wpAge* × *wpDS* (γ _12_)	0.01	0.02	–0.01	0.02	—	—	—	—
*wpAge* × *bpDS* (γ _13_)	0.02	0.01	0.00	0.01	—	—	—	—
*bpAge* × *wpDS* (γ _14_)	0.01	0.01	0.02*	0.01	—	—	—	—
*bpAge* × *bpDS* (γ _03_) EL or SL	–0.01	0.01	–0.01	0.01	—	—	—	—
*wpEL* (γ _11_)	—	—	—	—	–0.01	0.05	—	—
*bpEL* (γ _02_)	—	—	—	—	–0.13**	0.05	—	—
*wpAge* × *wpEL* (γ _12_)	—	—	—	—	0.02	0.02	—	—
*wpAge* × *bpEL* (γ _13_)	—	—	—	—	–0.02	0.01	—	—
*bpAge* × *wpEL* (γ _14_)	—	—	—	—	0.01	0.01	—	—
*bpAge* × *bpEL* (γ _03_)	—	—	—	—	–0.01	0.01	—	—
*wpSL* (γ _11_)	—	—	—	—	—	—	–0.03	0.04
*bpSL* (γ _02_)	—	—	—	—	—	—	–0.18**	0.05
*wpAge* × *wpSL* (γ _12_)	—	—	—	—	—	—	–0.01	0.01
*wpAge* × *bpSL* (γ _13_)	—	—	—	—	—	—	–0.03**	0.01
*bpAge* × *wpSL* (γ _14_)	—	—	—	—	—	—	0.01	0.01
*bpAge* × *bpSL* (γ _03_)	—	—	—	—	—	—	–0.02**	0.01
Covariates								
*W* (γ _04_)	0.06	0.61	–1.51	0.63	4.11**	0.69	3.87**	0.69
*wpAge* × *W* (γ _15_)	–0.07	0.19	0.16	0.22	0.18	0.19	0.13	0.19
*bpAge* × *W* (γ _05_)	0.25	0.14	0.12	0.14	–0.17	0.15	–0.19	0.15
*wpDS* × *W* (γ _16_)	0.03	0.05	0.12	0.06	—	—	—	—
*bpDS* × *W* (γ _06_)	0.03	0.05	0.03	0.05	—	—	—	—
*wpEL* × *W* (γ _16_)	—	—	—	—	–0.01	0.06	–	–
*bpEL* × *W* (γ _06_)	—	—	—	—	0.08	0.06	–	–
*wpSL* × *W* (γ _16_)	—	—	—	—	—	—	0.03	0.05
*bpSL* × *W* (γ _06_)	—	—	—	—	—	—	0.07	0.06
*E* (γ _07_)	–0.15	0.10	–0.35**	0.10	0.66**	0.11	0.64**	0.11
*wpAge* × *E* (γ _17_)	–0.05	0.03	–0.04	0.04	0.03	0.03	0.02	0.03
*bpAge* × *E* (γ _08_)	0.03	0.02	0.04	0.02	–0.04	0.02	–0.03	0.02
*wpDS* × *E* (γ _18_)	0.00	0.01	–0.01	0.01	—	—	—	—
*bpDS* × *E* (γ _09_)	–0.00	0.01	0.00	0.01	—	—	—	—
*wpEL* × *E* (γ _18_)	—	—	—	—	–0.00	0.01	—	—
*bpEL* × *E* (γ _09_)	—	—	—	—	0.01	0.01	—	—
*wpSL* × *E* (γ _18_)	—	—	—	—	—	—	–0.01	0.01
*bpSL* × *E* (γ _09_)	—	—	—	—	—	—	0.01	0.01
Random effects								
Var. intercept	66.23**	3.39	55.21**	4.40	100.80**	5.79	100.45**	5.77
Var. w-p age	—	—	0.34	0.37	0.12	0.25	0.11	0.25
Cov. intercept, w-p age	—	—	–0.00	0.94	5.24**	1.08	5.20	1.07
Residual variance	33.72**	1.36	47.05**	2.10	33.89**	1.51	33.86**	1.51
Variance accounted for								
w-p	.010		.045		.070		.073	
b-p	.009		.049		.078		.080	

*Notes*: *N* between 1,271 and 1,285 participants who provided 2,385 observations. Unstandardized estimates and standard errors presented. wp = within-person; bp = between-person. Emotional loneliness (EL), social loneliness (SL), and the Digit Symbol (DS) were T-standardized using baseline data of the entire sample (*M* = 50; *SD* = 10). Age was grant-men centered at age 73.26 years. Est. = estimate; Var. = variance; Cov. = covariance; w-p = within-person; b-p = between person; W = women; E = education.

**p < .01, *p < .05.

We also see evidence for sex and education effects such that women score more than one-third of a *SD* (4.11/3.87) better than men on the cognitive test, and those who are more educated report lower SL (–0.35) and score better on the cognitive test (0.64), but do not report lower EL (0.15). Being 1 *SD* above on education goes hand in hand with being about one-fifth of a *SD* above on cognitive performance [*SD* (E) = 3.03 in Table 1, so 3.03 * 0.64 in Table 2 = 1.94 ~ 2 = 1/5 of 10.51).

**Figure 1.**
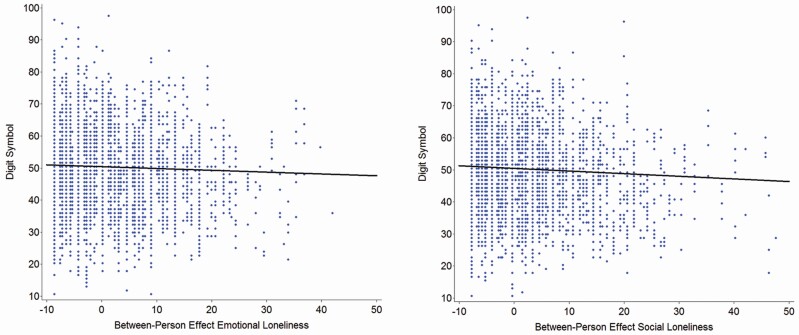
Illustrating between-person associations of emotional loneliness (left-hand panel) and social loneliness (right-hand panel) with the Digit Symbol test as a marker of perceptual speed. It can be obtained that loneliness discriminates individuals at the between-person level, such that those feeling emotionally or socially more lonely perform cognitively less well than those feeling emotionally or socially less lonely.

Most relevant for our research questions about the interplay between DS and loneliness, three findings are of note. First, there is no evidence whatsoever for predictive effects of the DS for age trajectories of EL or SL, either at the between-person level or at the within-person level. Second, there is also no evidence that EL or SL contribute to individuals’ decrease in cognition at the within-person level. Third, in contrast, predictive effects of loneliness for DS emerge at the between-person level, both for EL (–0.13) and SL (–0.18). As can be seen in Figure 1, loneliness discriminates individuals at the between-person level, such that those feeling emotionally or socially more lonely showed lower cognitive performance than those feeling emotionally or socially less lonely. The effect size of SL is roughly comparable to that of education, with 1 *SD* difference in SL being associated with about a fifth *SD* difference on the DS [*SD* (SL) = 10.12 in Table 1, so 10.12 * 0.18 in Table 2 = 1.82 ~ 2 = 1/5 of 10.51]. We also portrayed a locally weighted scatterplot smoothing (loess) relation between DS and the two loneliness variables, and in both cases obtained a virtual straight-line relationship, thereby validating the linear parametrization specified in the model of Equation 1.

**Figure 2.**
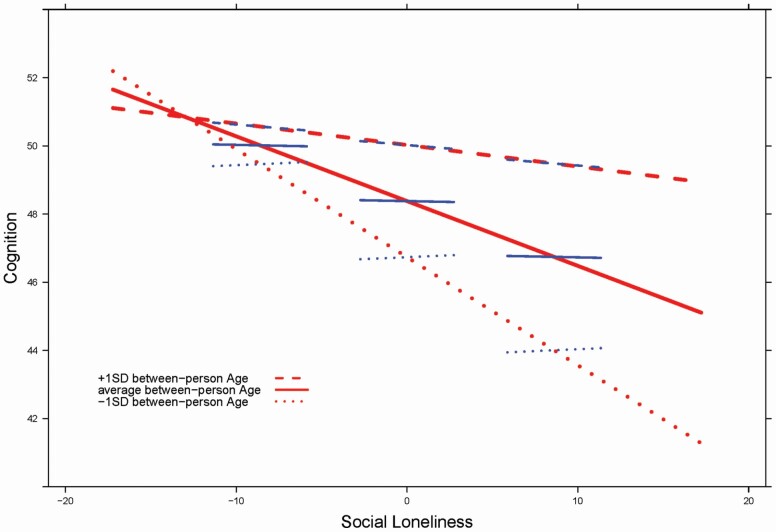
Illustrating between-person age associations (lighter red thick lines) and within-person age associations (darker blue thin lines) of social loneliness on perceptual speed. The continuous solid line refers to average between-person age, whereas the dashed and dotted lines refer to ±1 *SD* of age. It can be obtained that the predictive effects at the between-person level of social loneliness for (change in) cognition are stronger for people who are relatively younger in our sample (i.e., in their mid to late 60s) than for those who are relatively older in our sample (say in their 80s). The within-person social loneliness effects on the Digit Symbol, although being weak overall (all darker blue thin lines are close to flat), deviate from between-person social loneliness effects among those who are relatively younger in our sample (dotted lines), whereas they do not among those who are relatively older in our sample (dashed lines).

Finally, the between-person predictive effects of SL interacted with age both at the level of within-person (–0.03) and between-person effects (–0.02). To depict the various between-person and within-person effects of age and SL on DS, we ran a simplified version of the model that excludes the covariates (*W*_*i*_, *E*_*i*_) and their interactions. Figure 2 shows in lighter, red, thick lines the between-person age effects and in darker, blue, thin lines the within-person effects of SL on DS (for a similar depiction, see Thorvaldsson et al., 2012, 2020). The continuous solid line refers to the average between-person age value, whereas the dashed and dotted lines refer to ±1 *SD* of age, respectively. The negative slopes of all three thick red lines show the negative between-person effects of SL on the DS, and the fact that the dotted line is much steeper than the dashed line portrays the interaction of between-person SL effects and age. In other words, the predictive effects at the between-person level of SL for (change in) cognition are stronger for people who are relatively younger in our sample (i.e., in their mid to late 60s) than for those who are relatively older in our sample (i.e., in their early 80s). Moreover, Figure 2 shows that the within-person SL effects on DS, although being weak overall (all darker, blue, thin lines are nearly flat), deviate from between-person SL effects among those who are relatively younger in our sample (dotted lines), whereas they do not among those who are relatively older in our sample (dashed lines). This underscores the age effect on the contrast between within- and between-person effects of loneliness on cognition.

Following Snijders and Boskers (2012), we calculated the variances accounted for in our models by comparing the reduction in unexplained variance between the full model reported in Table 2 and an empty model that did not include any of the predictors. Results revealed (see bottom portion of Table 2) that about 1% of the variance both at the within-person level and the between-person level was explained in EL. For SL, these numbers were around 4.5% and 4.9%, and the explained variance for the DS was between 7% and 8%.

## Discussion

Our major objective was to disentangle age-related between-person from within-person associations between perceptual speed and loneliness in old age. Results revealed declines on perceptual speed and slight increases in EL with aging over time. We found no predictive effects of perceptual speed for age trajectories of EL or SL, neither at the between- nor at the within-person level. We also did not find evidence that SL or EL contributed to individuals’ decrease in cognition at the within-person level. However, both SL and EL predicted perceptual speed at the between-person level. Being in one’s 60s and early 70s was associated with stronger predictive effects of SL for perceptual speed and with a discrepancy in the direction and size of between-person and within-person associations.

### Age Trajectories of Cognitive Functioning and SL and EL in Old Age

Our results of within-person declines on perceptual speed by almost half a standard deviation across a 10-year epoch are consistent with a myriad of earlier findings reporting substantial decrements in fluid cognitive abilities (Salthouse, 2004, 2009; Tucker-Drob et al., 2019). Of note though is that the between-person effect of age was not statistically significant, indicating that those in their early 80s did not perform worse on the cognitive functioning test than those in their late 60s. This might be a result of the positive selection of the sample (particularly of those in older ages) and our participants’ relatively good health (König et al., 2018). Importantly, performance advantages for women over men and for those who are more educated corroborate the results of a recent meta-analysis on the role of education in adult cognition (Lövdén et al., 2020).

The average stability found in BASE-II for age-related loneliness trajectories also mirrors reports from the extant literature. For example, Mund and colleagues (2020) have concluded from the meta-analysis that mean levels of loneliness essentially remain stable from adolescence to oldest old age. It could be that the complex interplay of gain- and loss-related experiences in older age (e.g., loss of long-term friends vs better emotion regulation) cancel out one another. Our finding that more educated people report lower levels of SL, but not EL, also mirrors results from a meta-analysis (Pinquart & Sörensen, 2001) and may reflect the fact that more educated, and thus economically more privileged, individuals have more resources and overall opportunities to participate in social activities (e.g., clubs, restaurant visits) and also have more resources to actively regulate their social lives. This might not necessarily go hand in hand with the quality of those relationships and therefore is not reflected in EL.

### The Interplay Between Cognitive Functioning and Loneliness in Old Age

Most important for our research question, we can only speculate about why we did not find evidence for predictive effects of perceptual speed for age trajectories of EL or SL, either at the between-person or at the within-person level. It is possible that the predictive validity of perceptual speed for loneliness emerges later in life (e.g., in very old age or closer to death) when overall resources and functioning show more marked declines or have reached more impoverished levels. It is also possible that declines in cognitive abilities that are more salient (e.g., memory) are stronger predictors of withdrawing from social connections.

Our results on the predictive effects of both EL and SL for perceptual speed performance at the between-person level are in line with previous work (Donovan et al., 2017), highlighting the predictive validity of loneliness for cognitive functioning. That these associations did not exist at the within-person level suggests that this relationship holds across individuals, rather than within-persons. The presence of between-person level associations might reflect the operation of processes occurring earlier in the life span. Feeling emotionally and socially lonely compared to others might reduce one’s exposure to sensory and cognitive stimuli, which in turn may result in less stimulating environments (Wilson et al., 2007). That such associations were stronger for our participants in their 60s and early 70s could be taken to indicate that SL might be more detrimental for cognitive functioning in earlier phases of old age, when the normative expectation remains to be socially embedded into a larger network (Carstensen et al., 1999). Also, the finding that the direction and size of between-person and within-person associations differed among those in their 60s and early 70s, but not for older participants, suggests that factors contributing to within-person associations of perceptual speed and loneliness in younger-old individuals may not contribute to average between-person associations of perceptual speed and loneliness.

Corroborating previous research, we also found effects for sex and education such that women and those who were better educated performed better on the cognitive test. Those who were better educated also reported lower SL. Future mechanism-oriented studies need to examine pathways underlying such associations.

Based on our study design, we cannot draw inferences about underlying mechanisms. As one possible pathway, a number of studies have identified overlapping neuroanatomical substrates and shared brain regions of cognition and loneliness (see Düzel et al., 2019; Dolcos et al., 2020), including limbic brain regions and parietal and prefrontal cortices (i.e., cingulate, amygdala, and insula). Age- and pathology-related changes in such brain structures and functioning (e.g., brain atrophy) may thus contribute to across-domain associations of age-related changes in cognition and loneliness. As a second possible pathway, neurotransmitters have been shown to facilitate both cognitive function and lack of loneliness, and so may constitute neural correlates of cognition–loneliness changes and interactions. For example, the dopaminergic system is involved in regulating information processing, thereby enabling learning and memory consolidation processes, and also in decreasing loneliness when activated in rewarding situations and positive social experiences (Dolcos et al., 2020). Growing empirical evidence suggests that alterations in physiological systems may operate as one of the key pathways linking both domains among older adults (Düzel et al., 2019).

### Limitations and Outlook

In closing, we note limitations of our study design, measures, and sample. First, we acknowledge that our study design is not balanced, with more longitudinal observations for loneliness than for the DS. Thus, our study can be considered a rather conservative test of the predictive effects of loneliness for age trajectories in perceptual speed. Yet, the design may undermine our statistical power to test the within-person predictive effects of perceptual speed for age trajectories in loneliness. Future studies examining these associations would benefit from including more than four measurement points. We also note that the spacing between measurement points was not equal across waves. In follow-up analyses, we accommodated the point by including the time of assessment as an additional covariate in our analyses and obtained substantively identical results. However, future research would need to further examine whether or not the time interval between measurements might affect patterns of (within-person) associations. For example, it is an open question whether or not (within-person associations of) changes in perceptual speed and EL or SL occur at the same pace.

As limitations of our measures, we note that we had no information on more fine-grained indices of cognitive functioning or social functioning. This would have been highly informative because results might not generalize to other domains of cognitive (e.g., episodic memory; working memory) or social (e.g., network composition, quality of social interactions) functioning. To illustrate, based on previous research, one could assume that not social interactions per se but the quality of such interactions might be relevant to cognitive functioning in older age (Windsor et al., 2014). It could also be highly informative to examine potential moderators. For example, lifestyle factors such as (former) neighbors or marital status and type or exercise may not only help maintain cognitive functioning (Hertzog et al., 2008), but also protect against loneliness, because these factors provide a social context, cognitive–physical activities, and potentially a sense of belonging (e.g., to sports clubs or a group of colleagues). Similarly, mental health and, especially, depression have been linked to both loneliness and cognitive functioning (Sin et al., 2018). Future studies should examine the moderating effect lifestyle factors and mental health on the association between loneliness and perceptual speed.

For conceptual reasons (e.g., Mund et al., 2020) and reasons of parsimony, we decided to examine emotional and social faces of loneliness separately. To nevertheless examine SL and EL as simultaneous predictors, we conducted a follow-up analysis (Supplementary Table 2). It appears as if associations of SL with perceptual speed are more robust than those of EL. This may suggest that the perception of the quantity of one’s support network (i.e., friends, family, and neighbors) may be more important for processing speed. Future studies need to further examine the complex interplay of loneliness dimensions on perceptual speed.

We also acknowledge that within-person reliabilities of EL were less than ideal. At the same time, we refer to Nezlek (2017), who had argued that the usual trait-level standards (with the reliability of .60–.80 considered moderate and only substantial if greater than .80) should be relaxed when it comes to within-person reliability because, among other reasons, there are typically fewer items in within-person designs to reduce participant workload, and fewer items automatically mathematically lead to lower reliability. In addition, we applied MLM reliability estimation (Raudenbush & Bryk, 2002), which is more conservative because it accounts for unreliability better than what is typically done with Cronbach’s alphas, such as estimates applied to trait reliability. We believe that the lower reliability estimate for EL might be a reason for its weaker association with perceptual speed compared to SL.

As limitations of our sample, we acknowledge that our participants were in relatively good health and did not cover the very old segments of the population. No participant included in our analysis had dementia, as indexed by multiple cognitive screening instruments at baseline assessment (see Röhr et al., 2020). As a consequence, our results may not generalize to more disadvantaged or very old segments of the population because persons with low educational level and poor health and those living in institutions were underrepresented in our sample.

We also note that our findings might not generalize across historical time. To illustrate, perceptual speed has been shown to improve across historical time (e.g., Gerstorf et al., 2015), whereas findings regarding SL and EL are rather inconclusive (Hawkley et al., 2019; Hülür et al., 2016). Additionally, a number of studies suggest that positive historical trends in cognitive functioning are attenuated or even reversed in very old age (80s and older) and towards the end of life (see Drewelies et al., 2019; Gerstorf et al., 2020). It is thus an open question whether the dynamics observed for current cohorts of older adults (as those tested here in BASE-II) differ from those observed in earlier historical times (as those among same-aged adults two to three decades ago). For example, one could expect that with overall improved cognitive functioning, associations between loneliness and perceptual speed emerge later in the life span (e.g., when people reach their 80s and 90s).

Lastly, although these results provide some evidence for interrelations between loneliness and perceptual speed in old age, these effects emerged only in certain constellations and were small in size and should thus be interpreted with caution.

## Conclusions

Results from the present study suggest that perceptual speed and loneliness as two key domains of life are interrelated in old age. Specifically, perceptual speed did not predict EL or SL, at either the between- or within-person level. However, loneliness discriminated individuals at the between-person level, such that those feeling emotionally or socially more lonely showed lower cognitive performance than those feeling emotionally or socially less lonely. Our findings also suggest that insights about the direction and size of associations gained at the between-person level do not necessarily generalize to the within-person level. This provides impetus for more mechanism-oriented research at the within-person level so as to move towards informing targeted interventions in the future. Future research into the mechanisms underlying such differential associations or the lack of such associations at the within-person level promises to shed light onto central processes of successful aging.

## Supplementary Material

gbab180_suppl_Supplementary_Figure_S1

gbab180_suppl_Supplementary_Material

## Data Availability

The data can be requested from the steering committee of the Berlin Aging Study II. Further details about the procedure can be obtained at www.base2.mpg.de.
