# Supplementary figures and images for: Age Trajectories of Perceptual Speed and Loneliness: Separating Between-Person and Within-Person Associations
Source: J Gerontol B Psychol Sci Soc Sci. 2021 Nov 9;77(1):118–29. doi: 10.1093/geronb/gbab180 (PMC8755905; doi:10.1093/geronb/gbab180)

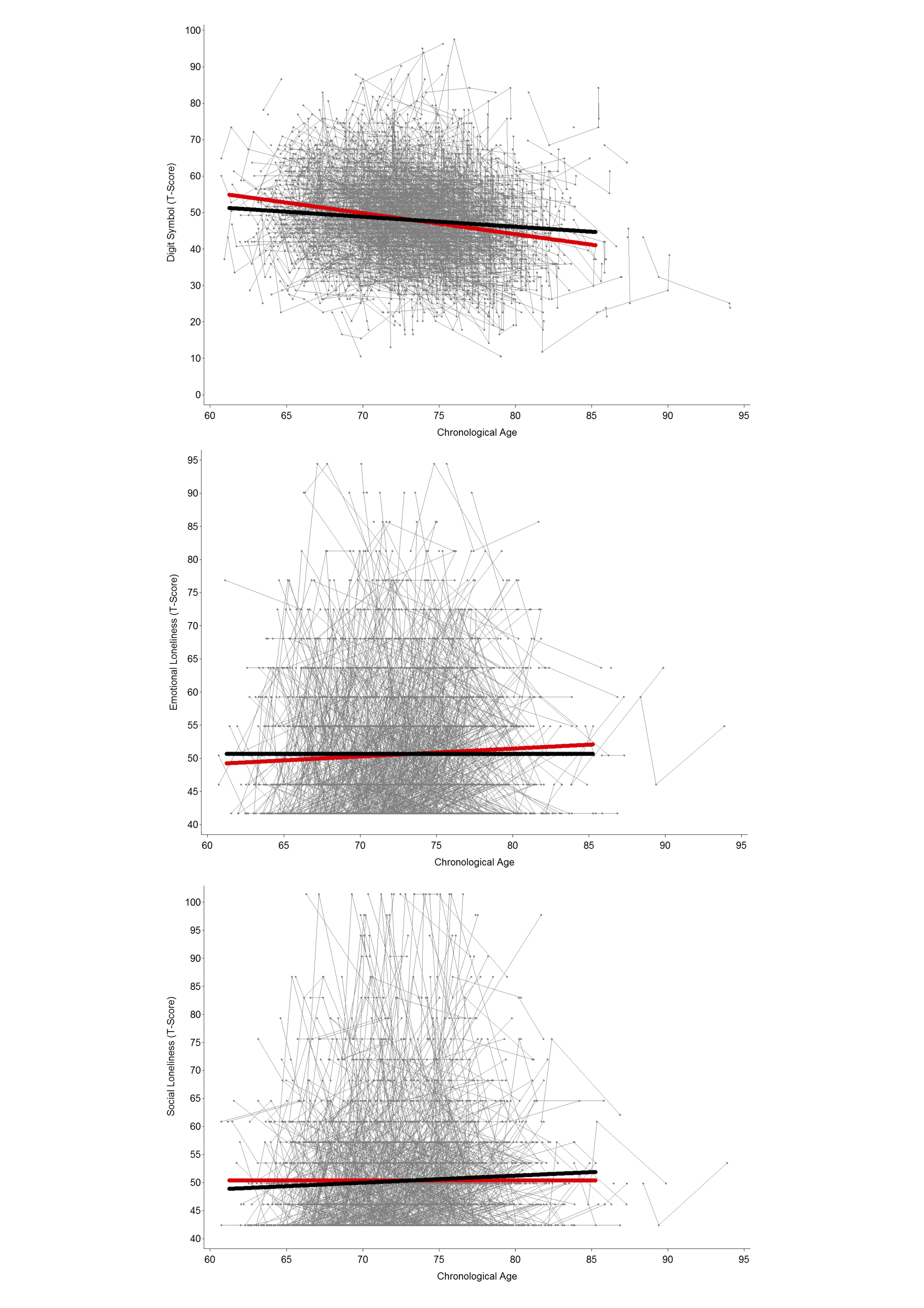

Supplement: gbab180_suppl_Supplementary_Figure_S1 [file gbab180_suppl_supplementary_figure_s1.jpeg]
