# Supplementary material for: Age Trajectories of Perceptual Speed and Loneliness: Separating Between-Person and Within-Person Associations
Source: J Gerontol B Psychol Sci Soc Sci. 2021 Nov 9;77(1):118–29. doi: 10.1093/geronb/gbab180 (PMC8755905; doi:10.1093/geronb/gbab180)
Supplement: gbab180_suppl_Supplementary_Material [file gbab180_suppl_supplementary_material.docx]

**Supplementary Online Material**

In our analyses, we tested for random effects of social and emotional loneliness, when predicting digit symbol; for the former, the model did not converge, whereas for the latter, all random effects about emotional loneliness were nonsignificant. Likewise, we tested for random effects of digit symbol when predicting both social and emotional loneliness. In both models, all random effects about digit symbol were nonsignificant.

Table SOM.1

*Descriptive Statistics for the Digit Symbol test, Emotional Loneliness, and Social Loneliness over Chronological Age.*

|  | Digit Symbol | | |  | Emotional loneliness | | |  | Social loneliness | | |
| --- | --- | --- | --- | --- | --- | --- | --- | --- | --- | --- | --- |
| Age | *n* | *M* | *SD* |  | *n* | *M* | *SD* |  | *n* | *M* | *SD* |
| 60 | 0 | – | – |  | 0 | – | – |  | 0 | – | – |
| 61 | 4 | 56.48 | 9.35 |  | 4 | 57.07 | 13.68 |  | 4 | 56.27 | 9.22 |
| 62 | 9 | 51.38 | 10.29 |  | 9 | 45.58 | 5.13 |  | 9 | 48.58 | 8.04 |
| 63 | 19 | 51.52 | 10.14 |  | 19 | 50.01 | 7.61 |  | 19 | 48.84 | 9.27 |
| 64 | 47 | 48.64 | 9.65 |  | 47 | 50.19 | 9.06 |  | 47 | 48.79 | 7.08 |
| 65 | 96 | 51.87 | 10.99 |  | 96 | 50.38 | 10.36 |  | 96 | 49.97 | 9.09 |
| 66 | 132 | 49.99 | 10.39 |  | 132 | 49.77 | 9.86 |  | 132 | 48.81 | 9.56 |
| 67 | 158 | 50.81 | 10.97 |  | 158 | 49.86 | 9.83 |  | 158 | 49.51 | 10.94 |
| 68 | 191 | 49.91 | 11.02 |  | 191 | 49.64 | 9.56 |  | 191 | 49.70 | 9.00 |
| 69 | 196 | 50.79 | 11.19 |  | 196 | 50.59 | 10.05 |  | 196 | 49.78 | 9.55 |
| 70 | 231 | 49.25 | 12.20 |  | 231 | 49.99 | 10.58 |  | 231 | 50.56 | 12.32 |
| 71 | 282 | 50.62 | 10.00 |  | 282 | 49.84 | 9.95 |  | 282 | 50.08 | 10.02 |
| 72 | 272 | 49.75 | 10.98 |  | 272 | 50.21 | 9.91 |  | 272 | 49.86 | 10.59 |
| 73 | 258 | 48.82 | 11.08 |  | 258 | 49.50 | 9.49 |  | 258 | 49.72 | 9.73 |
| 74 | 217 | 48.87 | 11.56 |  | 217 | 50.89 | 10.30 |  | 217 | 50.28 | 11.02 |
| 75 | 185 | 45.87 | 10.80 |  | 185 | 50.47 | 10.05 |  | 185 | 51.55 | 10.74 |
| 76 | 124 | 48.94 | 11.93 |  | 124 | 51.85 | 9.98 |  | 124 | 52.39 | 12.09 |
| 77 | 91 | 46.24 | 12.63 |  | 91 | 52.84 | 10.25 |  | 91 | 52.20 | 10.83 |
| 78 | 45 | 45.69 | 12.89 |  | 45 | 50.86 | 9.37 |  | 45 | 49.32 | 8.00 |
| 79 | 33 | 48.03 | 10.51 |  | 33 | 50.60 | 8.63 |  | 33 | 50.26 | 8.93 |
| 80 | 21 | 46.70 | 12.03 |  | 21 | 51.31 | 10.47 |  | 21 | 48.70 | 9.01 |
| 81 | 14 | 48.63 | 15.12 |  | 14 | 52.67 | 10.75 |  | 14 | 50.60 | 7.81 |
| 82 | 16 | 45.99 | 15.03 |  | 16 | 50.74 | 9.57 |  | 16 | 51.20 | 11.01 |
| 83 | 7 | 51.48 | 11.03 |  | 7 | 42.30 | 1.66 |  | 7 | 43.49 | 1.80 |
| 84 | 3 | 55.27 | 15.78 |  | 3 | 47.54 | 2.54 |  | 3 | 51.04 | 7.68 |
| 85 | 6 | 47.83 | 22.48 |  | 6 | 47.54 | 5.33 |  | 6 | 51.66 | 6.49 |
| 86 | 2 | 55.27 | 18.78 |  | 2 | 46.07 | 6.22 |  | 2 | 46.13 | 0.00 |
| 87 | 1 | 63.72 | – |  | 1 | 50.47 | – |  | 1 | 49.81 | – |
| 88 | 1 | 43.20 | – |  | 1 | 59.27 | – |  | 1 | 49.81 | – |
| 89 | 1 | 32.34 | – |  | 1 | 46.07 | – |  | 1 | 42.44 | – |
| 90 | 0 | – | – |  | 0 | – | – |  | 0 | – | – |

*Note*. *N* = 1,491 who provided 2,662 observations. T-scores standardized to cross-sectional
BASE-II sample at T1 (*M* = 50, *SD* = 10).

Table SOM.2
*Growth Models of Emotional Loneliness, Social Loneliness accounting for Subfacets of Loneliness*

|  | Digit Symbol | |
| --- | --- | --- |
|  | Est. | SE |
| **Fixed effects** |  |  |
| Intercept (γ*_00_*) | 46.48* | 0.47 |
| wpAge (γ*_10_*) | –0.47* | 0.12 |
| bpAge (*γ_01_*) | –0.12 | 0.09 |
| **Digit Symbol** |  |  |
| wpDS (*γ_11_*) | – | – |
| bpDS (*γ_02_*) | – | – |
| wpAge x wpDS (*γ_12_*) | – | – |
| wpAge x bpDS (*γ_13_*) | – | – |
| bpAge x wpDS (*γ_14_*) | – | – |
| bpAge x bpDS (*γ_03_*)  **EL and SL** | – | – |
| wpEL (*γ_11_*) | 0.02 | 0.05 |
| bpEL (*γ_02_*) | –0.02 | 0.07 |
| wpAge x wpEL (*γ_12_*) | 0.03 | 0.02 |
| wpAge x bpEL (*γ_13_*) | 0.00 | 0.02 |
| bpAge x wpEL (*γ_14_*) | 0.01 | 0.01 |
| bpAge x bpEL (*γ_03_*) | 0.00 | 0.01 |
| wpSL (*γ_11_*) | –0.03 | 0.04 |
| bpSL (*γ_02_*) | –0.16 ^a^ | 0.07 |
| wpAge x wpSL (*γ_12_*) | –0.01 | 0.02 |
| wpAge x bpSL (*γ_13_*) | –0.03 ^a^ | 0.02 |
| bpAge x wpSL (*γ_14_*) | 0.01 | 0.01 |
| bpAge x bpSL (*γ_03_*) | –0.02 | 0.01 |
| **Covariates** |  |  |
| W (*γ_04_*) | 3.92* | 0.69 |
| wpAge x W (*γ_15_*) | 0.15 | 0.19 |
| bpAge x W (*γ_05_*) | –0.19 | 0.15 |
| wpDS x W (*γ_16_*) | – | – |
|  |  |  |
| bpDS x W (*γ_06_*) | – | – |
| wpEL x W (*γ_16_*) | –0.03 | 0.06 |
| bpEL x Women (*γ_06_*) | 0.06 | 0.08 |
| wpSL x W (*γ_16_*) | 0.03 | 0.05 |
| bpSL x W (*γ_06_*) | 0.03 | 0.09 |
| E (*γ_07_*) | 0.65* | 0.11 |
| wpAge x E (*γ_17_*) | 0.03 | 0.03 |
| bpAge x E (*γ_08_*) | –0.04 | 0.02 |
| wpDS x E (*γ_18_*) | – | – |
| bpDS x E (*γ_09_*) | – | – |
| wpEL x E (*γ_18_*) | 0.00 | 0.01 |
| bpEL x E (*γ_09_*) | 0.01 | 0.01 |
| wpSL x E (*γ_18_*) | –0.01 | 0.01 |
| bpSL x E (*γ_09_*) | 0.01 | 0.01 |
| **Random effects** |  |  |
| Var. intercept | 100.61* | 5.77 |
| Var. w–p age | 0.10 | 0.25 |
| Cov. intercept. w–p age | 5.24* | 1.08 |
| Residual variance | 33.16* | 1.67 |
| Variance accounted for |  |  |
| wp | .070 | |
| bp | .078 | |

*Note*. *N* 1.495 participants who provided 2.662 observations. Grip = grip strength. emo lone = emotional loneliness. soc lone = social loneliness. Unstandardized estimates and standard errors presented. Emotional loneliness. social loneliness. and the Digit Symbol were T–standardized using baseline data of the entire sample (*M* = 50; *SD* = 10). Age was grant–men centered at age 73.26 years.
* *p* < .01. ^a^ *p* < .05

*Figure SOM.1*. Illustrating between-person age associations (lighter red trajectories) and within-person age associations (darker black trajectories) along with the individual (thin lines) trajectories for the Digit Symbol test as a marker of perceptual speed (upper Panel), emotional loneliness (middle Panel), and social loneliness (lower Panel). It can be obtained that perceptual speed exhibited on average moderate age-related declines, whereas both facets of loneliness were on average rather stable. The figure also highlights the tremendous amount of between-person heterogeneity in these age trajectories.
